# Proteinoid Polymers and Nanocapsules for Cancer Diagnostics, Therapy and Theranostics: In Vitro and In Vivo Studies

**DOI:** 10.3390/jfb14040215

**Published:** 2023-04-11

**Authors:** Ella Itzhaki, Yuval Elias, Neta Moskovits, Salomon M. Stemmer, Shlomo Margel

**Affiliations:** 1 Department of Chemistry and Institute of Nanotechnology and Advanced Materials, Bar-Ilan University, Ramat-Gan 5290002, Israel; elaeli3543@gmail.com (E.I.);; 2 Felsenstein Medical Research Center, Petah Tikva 49100, Israel; 3 Sackler Faculty of Medicine, Tel Aviv University, Tel Aviv 6997801, Israel

**Keywords:** proteinoid polymers, proteinoid nanocapsules, fluorescent nanocarriers, cancer theranostics, RGD nanoparticles

## Abstract

Proteinoids—simple polymers composed of amino acids—were suggested decades ago by Fox and coworkers to form spontaneously by heat. These special polymers may self-assemble in micrometer structures called proteinoid microspheres, presented as the protocells of life on earth. Interest in proteinoids increased in recent years, in particular for nano-biomedicine. They were produced by stepwise polymerization of 3–4 amino acids. Proteinoids based on the RGD motif were prepared for targeting tumors. Nanocapsules form by heating proteinoids in an aqueous solution and slowly cooling to room temperature. Proteinoid polymers and nanocapsules suit many biomedical applications owing to their non-toxicity, biocompatibility and immune safety. Drugs and/or imaging reagents for cancer diagnostic, therapeutic and theranostic applications were encapsulated by dissolving them in aqueous proteinoid solutions. Here, recent in vitro and in vivo studies are reviewed.

## 1. Introduction

Proteinoids are random polymers composed of amino acids synthesized by stepwise thermal polymerization. They were discovered and studied in the 1950s by Fox and coworkers, who suggested that they formed spontaneously by high heat at the beginning of life on Earth. Fox et al. demonstrated that proteinoids may self-assemble in spherical micrometer structures, proteinoid microspheres, presented as the protocells of life [1,2,3]. This process occurs at rather high temperature (170–180 °C, for instance) in an inert atmosphere without a catalyst or a solvent. Lysine or aspartic/glutamic acid are critical, as they form cyclic products which act as solvents [4]. Various proteinoids may be prepared by using natural and synthetic amino acids at different ratios. The special features of each proteinoid influence the character of particles that are composed from it [5,6].

In early work by Rao and coworkers [7,8], an acidic proteinoid was prepared by thermal condensation of seven natural amino acids, mainly Glu, Asp and Gly [7]. The proteinoid was expected to be non-antigenic due to its low molecular weight (<10 kDa). The acidic gastric irritating drug methotrexate was encapsulated in self-assembled microspheres rather efficiently (~50% with ~10% loading) [8], conferring gastric (pH~1) stability (<10% released in two hours) and thus improving the prospects of oral delivery. The spherical microspheres were of uniform diameter (1–3 μm). Complete solubility in neutral blood pH allowed release of most of the encapsulated drug within one hour.

During the last decade, Margel and coworkers produced proteinoids by bulk stepwise polymerization of 3–4 amino acids without or with a biopolymer such as poly(L-lactic acid) (PLLA) [9,10,11,12,13,14]. Nanocapsules (NCs) form spontaneously by heating proteinoids to about 70 °C in an aqueous solution to completely dissolve the polymers, followed by slow cooling to room temperature. Different drugs and/or imaging reagents were encapsulated by dissolution in the proteinoid solution, and covalent binding to the surface of the NCs was achieved with or without a spacer arm. Proteinoid NCs suit many applications owing to their non-toxicity, biocompatibility and immune safety [10,11]. Incorporation of the tri-amino acid sequence arginine–glycine–aspartic acid (RGD) in the random proteinoid backbone enables targeting of tumors.

Kwon, Park & Kim [15] demonstrated that proteinoids could also act as carriers of a certain kind of drugs and be programmed to release them depending on the external conditions. This is made possible by introducing a disulfide bond in the proteinoid that lets it assemble in an aqueous phase. In an external environment which is reducing in nature, the disulfide bond is cleaved, and the micelle is likely to be loosened. As a result, the payload (drug) inside the micelle would be released.

Recent work by Adamatzky investigated the potential application of proteinoids in the field of computing systems [16]. Proteinoids have the potential to be utilized in unconventional computing owing to their unique electrical properties. Such proteinoids were also prepared by step-growth polymerization of amino acids in an aqueous environment. These proteinoids swell into hollow microspheres that produce an endogenous burst of electrical potential spikes and exhibit the capacity to alter patterns of their electrical activity in response to illumination. By forming interconnected networks through pores and tubes, proteinoid microspheres can enable programmable growth and have potential for creation of intricate computing systems. This capacity for novel growth patterns and electrical activity allows for the possibility of developing computing architectures that are more versatile and efficient than traditional systems.

The present review article builds upon the foundation established by Kolitz-Domb and Margel in 2018 [11] that focused primarily on the chemistry behind the preparation of a few proteinoids and their nanocapsules (e.g., P(KF), P(EF) in the absence or presence of PLLA), as well as their application in select fields such as cosmetics, agriculture and theranostics using the cancer drug doxorubicin. This review describes mainly the synthesis and characterization of P(RGD) polymers and nanocapsules containing synergistic drugs, cannabinoids and TRAIL for cancer theranostics. The main focus is on P(RGD) application in the realm of cancer research. Our aim is to provide an up-to-date in vitro and in vivo survey of the latest findings in this rapidly evolving field, shedding light on new developments and offering fresh insights into the potential of proteinoid nanocapsules for cancer diagnostic, therapeutic and theranostic applications, including targeted drug delivery systems.

## 2. Synthesis and Characterization of Proteinoids and NCs

### 2.1. Preparation of Proteinoids

**Figure 1:**
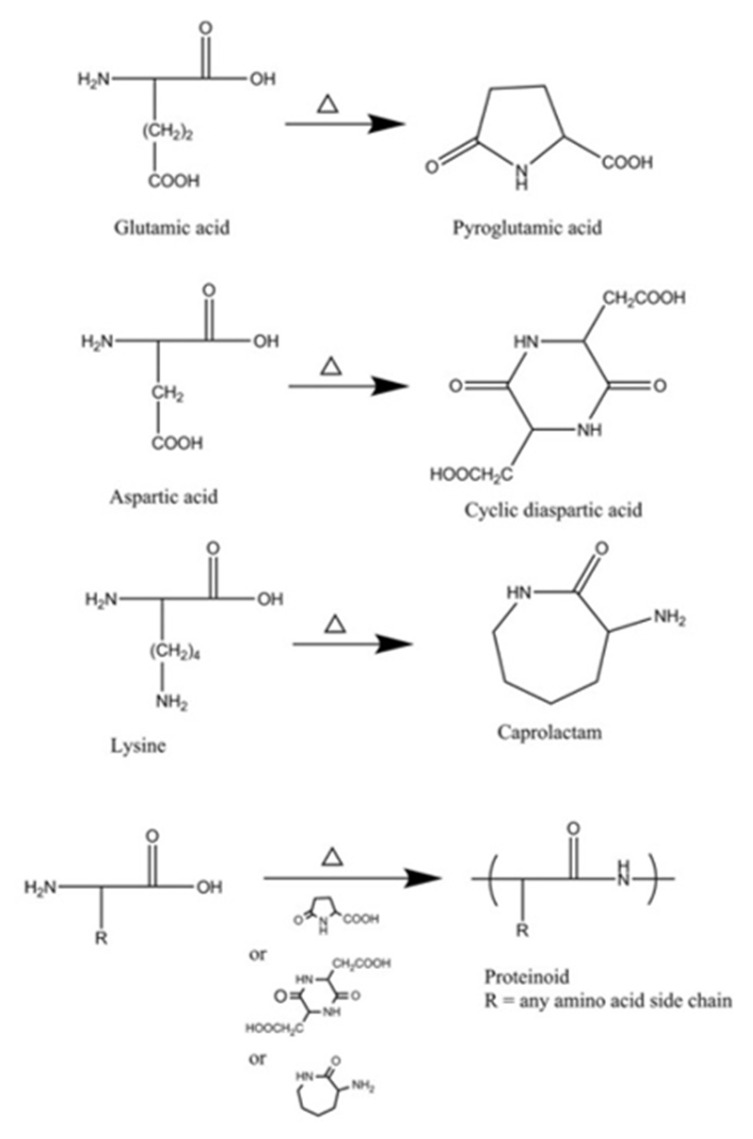
Proteinoid formation by polymerization with heat in a cyclic form of Lys/Asp/Glu as solvent [11].

Proteinoid chains were prepared by step-growth polymerization of amino acids at high temperature (depending on the amino acids, e.g., 180 °C) in an inert atmosphere with no solvent, initiator or catalyst [11,12]. A tri-functional amino acid—Glu/Asp/Lys—is an essential component, providing a solvent by cyclization and serving as an initiator (see Figure 1) [11]. Proteinoid preparation was recently reviewed [17]. Different proteinoid polymers can be obtained due to the extensive range of both natural and synthetic amino acids available (in most cases, the ratio between the monomers was 1:1). This review presents several examples of proteinoid polymers that have been studied for the purpose of cancer diagnostics, therapy and theranostics (see Table 1).

### 2.2. Preparation of Nanocapsules (NCs)

**Figure 2:**
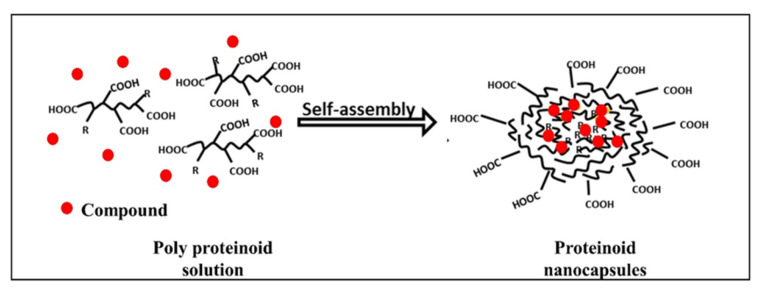
Self-assembly of proteinoid polymers; scribbled lines—polymer chains, dots—encapsulated compound (dyes/drugs) [11].

Hollow proteinoid NCs were produced by a self-assembly process in an aqueous continuous phase [17]. In this process, the first step was to heat the aqueous phase containing the proteinoid to about 70–80 °C until a full dissolution of the polymer was observed, followed by slow cooling to room temperature for precipitation and formation of proteinoid NCs. The NCs form biocompatible carriers with a hydrophobic core and hydrophilic groups on the surface. Various compounds were encapsulated during self-assembly, as presented in Figure 2. Near infrared (NIR) fluorescent dyes were used for cancer diagnosis [12,17,18]. Recently, a synergistic combination of anti-cancer drugs was encapsulated [19] for personalized therapy.

### 2.3. Characterization of Proteinoids and NCs

**Figure 3:**
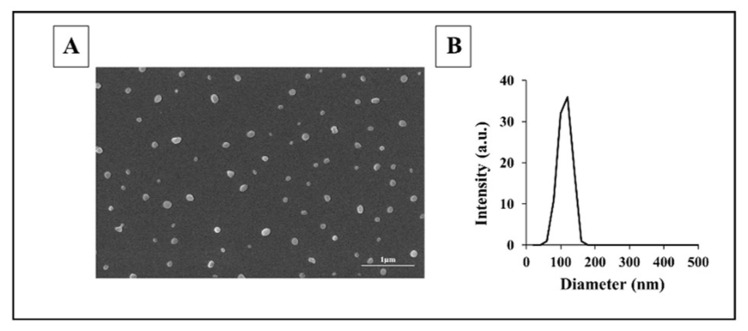
SEM image (**A**) and diameter histogram (**B**) of proteinoid nanocapsules (NCs) [19]. The diameters of more than 200 NCs were measured with Analysis Auto image analysis software version 3.2 (Soft Imaging System GmbH, Münster, Germany).

Unexpectedly, the molecular weight of all proteinoids made in the Margel lab was high (25–195 kDa) with a low polydispersity index (PDI of 1.01–1.27) [5]. These very unusual results for stepwise polymerization may be explained by the high temperature, which provides uniform long chains that resemble natural biopolymers. Self-assembled NCs were spherical with a uniform distribution, as shown by dynamic light scattering (DLS) and scanning electron microscopy (SEM) in Figure 3.

## 3. Cancer Diagnostics and Therapy, towards Theranostic Applications

Over the past decade, various proteinoids and nanocapsules (NCs) were synthesized in the Margel lab. In this section, we provide a brief overview of early applications, which were previously discussed in Kolitz-Domb’s 2018 review [11] that focused on detection of colorectal cancer (CRC). We then present new developments in cancer diagnostics and therapy towards theranostic applications. Recently, theranostics, a field that combines therapy and diagnostics, has gained considerable attention. Compared to targeted conjugates of peptides with drugs/agents, theranostics offers significant advantages [20].

### 3.1. Colorectal Cancer (CRC) Diagnostics

The first application of proteinoids studied by Margel and coworkers aimed at early specific detection of colorectal cancer (CRC) [14,17]. Indocyanine green (ICG), a fluorescent dye in the NIR region, 700–1000 nm [21,22], that is FDA-approved was encapsulated in biodegradable proteinoid NCs composed of Glu and Phe with PLLA [10,14]. Peanut agglutinin (providing targeting) and anti-caci-nonembryonic antigen antibodies (termed anti-CEA) were attached [23]. These as well as hollow P(EF-PLLA) NCs allowed specific detection of CRC tumors in chicken embryo tumor implants. The high signal in tumors was attributed to receptor upregulation [14].

**Figure 4:**
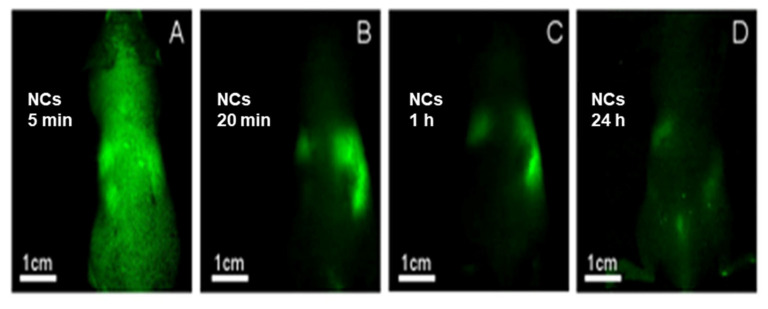
Whole-body images of NIR fluorescent P(EF-PLLA) NCs after intravenous (IV) injection to mice at (**A**) 5 min, (**B**) 20 min, (**C**) 1 h and (**D**) 24 h [14].

**Figure 5:**
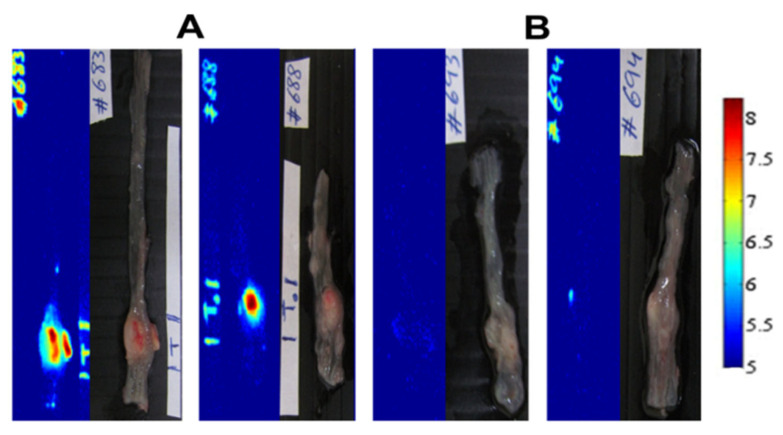
Specific targeting of colon tumors—P(EF-PLLA) NCs with anti-CEA (**A**) vs. IgG (**B).** Fluorescent (**left**) and grayscale (**right**) images [14].

ICG encapsulation was optimized, and spherical particles were obtained with wet/dry diameters of 145 ± 20/70 ± 15 nm. The encapsulation increased photo-stability significantly, protecting the dye from light-induced bleaching. The NCs were non-toxic as expected, also at a high concentration of 2.5 mg/mL. Interestingly, biodistribution in mice (after injection) showed complete clearance after 24 h from various locations including the brain and bones (Figure 4). This is useful for targeted biological applications. Specific detection of colon tumors by the fluorescent NCs was demonstrated in chicken embryos and mice (Figure 5), offering a significant advantage over invasive colonoscopy. The anti-CEA-conjugated NCs specifically detected tumors in mice with a specific signal (reporting signal to noise ratios) (Figure 5A). On the other hand, no signal was obtained with NCs attached to an anti-rabbit non-selective antibody (see Figure 5B) [14].

### 3.2. Cancer Therapy with Doxorubicin-Loaded NCs

Kiel et al. used proteinoid NCs to encapsulate the anticancer compound doxorubicin (Dox) [10]. The NCs showed similar activity as the anticancer drug Doxil, comprising another nanocarrier, a polyethylene glycol (PEG) lipid surface that prolongs blood circulation and improves tumor uptake [24]. The side effects of Dox were avoided by sufficient encapsulation. Four basic L-amino acids were selected, along with PLLA; lysine was the main monomer, enabling the synthesis. A series of P(KRHF-PLLA) proteinoids with varying His/Phe ratios were prepared with very low PDI (1.01–1.03) and high molecular weight (122–149 kDa).

Particle diameter was optimized. A 2:3 His/Phe weight ratio produced the smallest hollow particles (36.2 ± 6.9 nm). The corresponding Dox-loaded particles were 112 ± 15 nm (by DLS). The nanometric diameter is important for cancer therapeutics, allowing particles to cross biological barriers, penetrate cells and evade immunogenic systems [25]. The NCs exhibited a narrow diameter distribution (PDI~1), an important characteristic for biomedicine. Drug content was optimized at 15% Dox (compared to 12.5% in Doxil) [14]. Successful encapsulation was confirmed by FTIR (93 ± 12%) and XRD (amorphous vs. crystalline phase). The diffractogram of free Dox shows clear peaks, indicating a crystalline phase, while the proteinoid shows an amorphous pattern. The XRD pattern of Dox-loaded NPs shows a reduction in peak number and intensity compared to free Dox, indicating a phase transformation from crystalline to amorphous doxorubicin [10].

PEG was conjugated to enhance stability and prevent drug leakage [26]. PEGylated Dox-loaded NCs were shown to be more stable when changing the environment, and the release of the drug was confirmed to occur only in blood, while non-PEGylated NCs also release it in serum and phosphate-buffered saline (PBS). This selective release becomes important when considering long-term shelf life, ensuring release only upon exposure to proteolytic enzymes and other blood ingredients.

**Figure 6:**
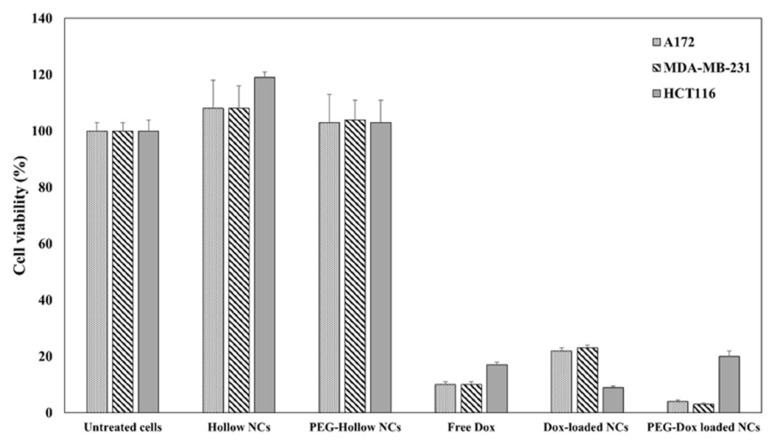
Viability of HCT116 colon carcinoma, ductal carcinoma and A172 glioblastoma cells 48 h after treatment with Doxil vs. Dox-loaded P(KGHF-PLLA) NCs, measured by XTT assay [10]. The cells were exposed to NCs at a concentration of 0.05 mg/mL, as well as to an equal quantity of free Dox.

An in vitro colorimetric assay (Figure 6) reveals a similar cytotoxic activity as free Dox of NCs and even higher toxicity of PEGylated NCs toward glioblastoma and ductal and colon carcinoma cells. The higher dose of loaded NCs, i.e., 0.05 mg/mL (drug loading: Dox concentration of 8.3 µg/mL), exceeded free Dox as shown in Figure 6.

## 4. Cancer Theranostics

Proteinoid NCs may encapsulate other drugs such as Taxol and Temozolomide and provide cancer theranostics by co-encapsulation of an anticancer drug and a NIR fluorescent dye. Moreover, conjugation of suitable targeting agents such as TRAIL to PEGylated Dox-loaded NCs is likely to enhance delivery and increase penetration rate [12].

### 4.1. RGD (ArgGlyAsp) for Specific Delivery to Tumors

The RGD motif was discovered in 1985 by Pierschbacher and Ruoslahti as the active component in fibronectin [27]. This peptide has high affinity to αvβ3 integrin, which is overexpressed in cancer cells and upregulated on the surface of growing blood vessels and is thus attracted to areas of angiogenesis, affording integrin-targeted nanodrugs for tumor imaging and treatment [28,29,30].

Both cyclic and linear RGD peptides were conjugated to NCs for targeted delivery [31]. The optical activity, rotating polarized light to the left (L) or right (D), influences attachment to cells. For example, replacing L-Asp with the D isomer yields an inactive peptide, while D-Arg increases binding 10-fold [31,32]. However, recent studies of biomedical applications do not emphasize this aspect [33,34,35].

Optimal RGD configuration offers significant improvement. Hadad and coworkers recently prepared novel RGD proteinoid polymers and NCs [13]. Different configurations were used to randomly achieve the RGD sequence in about 13% [36] of the proteinoid backbone. Such P(RGD) proteinoid NCs may act both as a nanocarrier and as a targeting system due to the RGD motif. This work suggests a rapid method for economic synthesis of proteinoid NCs for therapeutic, diagnostic and theranostic cancer applications.

### 4.2. Preparation and Characterization of RGD Proteinoids

To determine optimal RGD configuration for targeted delivery, four proteinoid configurations were synthesized: P(R^D^/RGD^D^/D). Asp serves as solvent and as a linker that reduces the energy of polymerization. In contrast to previous publications, cross-linked products were not obtained, and all four configurations were obtained with 100% yield and were water soluble. The proteinoids had molecular weights of 67,660−69,066 Da, a very low PDI [10,11,12] atypical of stepwise polymerization, which usually yields light polymers with high PDI [37].

### 4.3. Self-Assembly and Characterization of P(RGD) NCs

Hollow P(RGD) NCs were formed by self-assembly of the crude proteinoid by heating the solution and cooling slowly to room temperature. The diameters as determined by SEM were all similar at 55 ± 13, 48 ± 9 and 42 ± 9 nm for R^D^ and D^D^, R^D^, and D^D^, respectively, and 45 ± 11 nm for P(RGD). ICG was successfully encapsulated with dry diameters of 141 ± 24 nm for R^D^ and D^D^, 95 ± 13/87 ± 12 nm for R^D^/D^D^ and 86 ± 11 nm for P(RGD). The capsules thus swell considerably [38].

### 4.4. ICG Controlled Release

**Figure 7:**
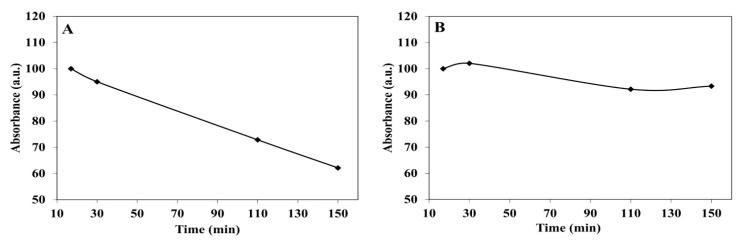
Absorbance of ICG-loaded P(R^D^GD) NCs in human serum (**A**) vs. PBS (**B**) at 37 °C over 2.5 h [13].

Drug release is commonly studied in vitro by incubation in serum or PBS [39]. ICG release was evaluated in physiological conditions by incubating the NCs for 2.5 h at 37 °C. The kinetics of ICG release from P(R^D^GD) NCs after treatment in serum/PBS is shown in Figure 7. As expected, there is little release to PBS, while in serum, peptide bonds are cleaved and ICG is significantly released.

### 4.5. Optimization of RGD Configuration

Angiogenesis, a cascade of cellular events in which new blood vessels are developed, is essential in many conditions including psoriasis and cancer [40]. Human vascular endothelial cells (HUVECs) interact with the environment using receptors known as integrins, which regulate growth/repair of blood vessels [40]. The high affinity of RGD to αvβ3 integrin attracts it to angiogenesis areas [27,29,41,42,43].

**Figure 8:**
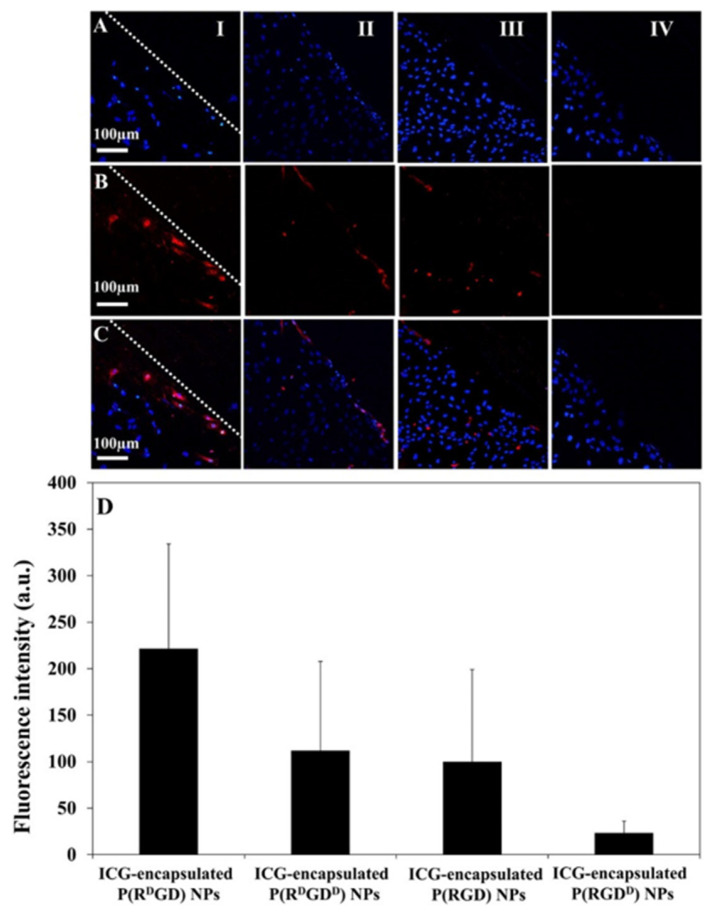
Scratch test assay [13]. HUVEC after treatment with ICG-loaded P(RGD) NCs show lines of nuclei with blue Hoechst dye (**A**) or red NCs (fluorescent, (**B**)) as well as in overlay images (**C**). Scale bar 100 μm (I–IV for R^D^GD, R^D^GD^D^, RGD and RGD^D^). Quantification of fluorescence intensity of scratch region images, analyzed by ImageJ 1.52a (**D**).

A scratch assay commonly employed for studying angiogenesis in vitro [39,44] was applied to find the optimal NC configuration. Confocal images of HUVECs after short (15 min) incubation with ICG-loaded NCs (Figure 8A–C) show clear accumulation in the injured scratch zone (red). The strongest signal is exhibited by P(RGD^D^) NCs. The fluorescence in the scratch zone (Figure 8D) clearly shows significantly higher intensity for P(R^D^GD)-treated cells, in accordance with the high affinity of D-Arg to integrins expressed on the cell membrane. Hence, cell attachment appears to be negatively affected by D-Asp.

These results further suggest that the optical configuration directly influences the biological activity [29,31]. P(R^D^GD) NCs with the strongest signal in the scratch zone are hence the best candidates for targeted carrying of drugs to angiogenesis areas such as wounds or tumors.

### 4.6. Engineering of NIR Fluorescent PEGylated P(R^D^GD) NCs

The NIR dye ICG was loaded within P(R^D^GD) NCs during self-assembly of the proteinoid NCs. The dry diameter increased with ICG from 47 ± 9 to 95 ± 1 nm [37]. ICG-bearing NCs were PEGylated to improve stability. PEGylation was found to be important for drug delivery, resulting in improved blood circulation, [45] phagocytosis evasion [46] and better stability in serum [47]. The latter is critical for prevention of dye/drug leakage [48,49]. On the other hand, antibodies may form which specifically recognize and bind PEG [47]. The attachment of PEG (Mw 750 or 5000) to the ICG-encapsulated P(R^D^GD) NCs was done by conjugation of NHS ester groups (NHS-PEG) to the primary amine group of the NCs. The hydrodynamic diameter of the loaded NCs, 93 ± 20 nm, increased following PEGylation with NHS-PEG, 750 or 5000 Da, to 177 ± 30 and 216 ± 25, respectively.

To evaluate the release of ICG, the NCs were incubated for 2.5 h in physiological conditions (PBS or serum at 37 °C). The absorbance of ICG was measured by UV spectroscopy. The sustained release in serum probably results from proteases [12]. Clearly, after incubation in serum, the absorbance of the NCs without PEG decreased significantly, by 40%, while there was a significantly lower decrease in absorbance with 5000 and 750 Da PEG, 10% and 20%, respectively. This difference was attributed to the PEGylation, which prevents biodegradation; the longer the PEG chain, the higher the stability of encapsulated ICG [50]. The results clearly demonstrate the successful PEGylation as well as the major effect of PEG on the rate of ICG release from P(R^D^GD) nanocapsules.

## 5. Application of P(RGD) NCs for Cancer Tumors

### 5.1. mCherry-Labeled Tumor in CAM Model

The well-known chick chorioallantois membrane (CAM) experiment is a simple, cost-effective animal model that avoids animal suffering due to the use of embryonic tissue. The highly vascularized membrane around the embryo allows grafting of tumor explants, [51] enabling rapid in vivo studies with fewer mice required later.

**Figure 9:**
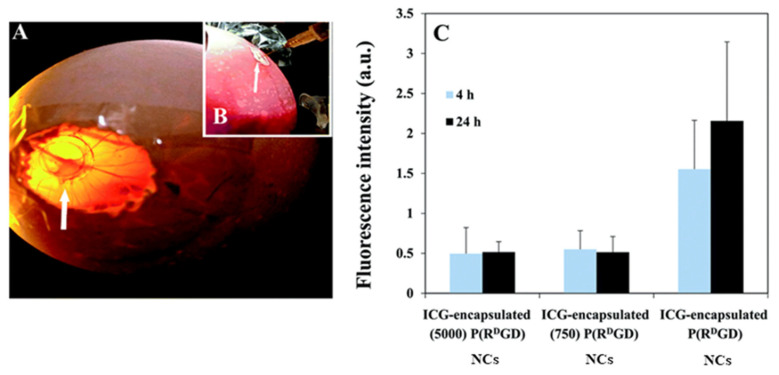
Tumor on CAM (top view, (**A**)) and P(R^D^GD) NCs injected IV (**B**). Fluorescence from implanted carcinoma cells with encapsulated ICG 4/24 h after the injection (**C**) [36].

A CAM window was exposed after incubation of fertile eggs, and implanted cells began to grow on a ring made of plastic (Figure 9A). ICG-loaded P(R^D^GD) NCs with or without PEG were injected IV (Figure 9B), and mammary carcinoma explants were examined after 4/24 h (Figure 9C) [13].

The non-PEGylated NCs showed significantly higher fluorescence compared to the PEGylated ones, indicating greater accumulation of ICG. This is in accord with the better packing with PEG with fewer bonds that may be bio-degraded, which significantly slows the release by preventing quick disassembly of the NCs at the tumor site.

### 5.2. mCherry-Labeled Tumor in Mouse Model

The targeting of the ICG-encapsulating P(R^D^GD) NCs toward tumors for a prolonged duration (beyond 24 h) was evaluated in vivo using a xenograft model [36]. Balb/c mice were injected with mouse carcinoma cells subcutaneously, inducing a xenograft. After two weeks, the mice were treated IV with ICG or P(R^D^GD) NCs loaded with ICG (with or without PEG) [13].

**Figure 10:**
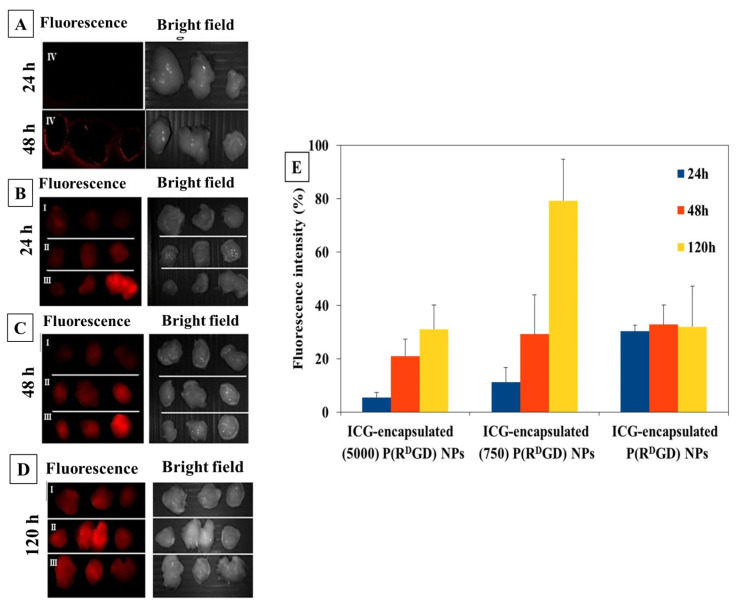
Images of P(R^D^GD) NCs with encapsulated ICG with long (5000 Da, I) and short (750 Da, II) PEG chains and without PEG (III) toward carcinoma tumors after (**B**–**D**) 24/48/120 h and ICG (IV) after (**A**) 24/48 h. Tumor fluorescence intensity after NC treatment (**E**) [36].

The fluorescence of tumors harvested after 24/48/120 h (Figure 10) shows that as time progresses, the NCs remain visible. This clearly indicates a targeted delivery (Figure 10A–C). For ICG, the fluorescence rapidly decreases and after 24 h is absent in the areas of the tumor (Figure 10D). The major role of PEG in stabilization is clearly seen; the fluorescence increases with time, while without PEG, it decreases (Figure 10E).

The targeting of the NCs is affected by the PEG chain length. The migration toward the tumor is reduced with the longer chain, indicating enhanced shielding [52]. After 120 h, the short PEGylated NCs were the most efficient. Recent studies [53,54] suggest that small uncharged NCs may be ideal for targeted delivery, as they interact less with cells. Accordingly, the short PEGylated NCs are smaller than the long PEGylated ones (177 ± 30 vs. 216 ± 25 nm) and essentially uncharged (5 ± 3 mV); without PEG, the charge is quite significant (30 ± 7 mV).

### 5.3. In Vivo Anti-Tumor Therapy with Cannabidiol (CBD)

Polymeric and other NCs were developed to improve the antitumor activity of cannabinoids [55]. Lugasi and coworkers recently presented CBD-loaded NCs designed for targeting human tumors [9]. P(R^D^GD) acidic proteinoids were prepared, and hollow/CBD-loaded NCs were formed with 97 ± 4/86 ± 3 nm diameter.

HPLC confirmed successful loading, and drug loading analysis found complete encapsulation. After lyophilization with trehalose and prolonged storage, redispersed particles retained their original diameter. Hollow NCs were found to be nontoxic, similar to CBD, and reached the cytoplasm. However, encapsulated CBD showed high toxicity, indicating efficient penetration and destruction of tumor cells.

**Figure 11:**
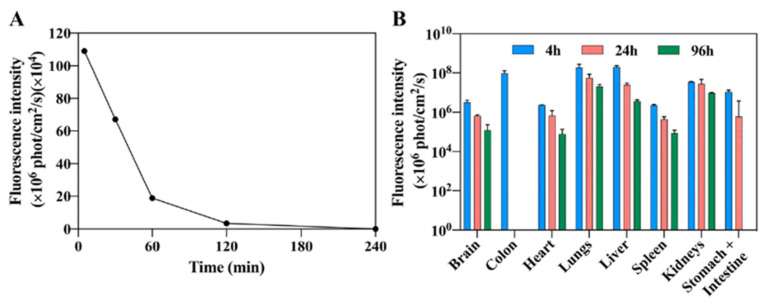
Blood fluorescence (**A**) and biodistribution (**B**) of Cy7-conjugated P(RGD) NCs [9]. Using the Maestro II in vivo imaging system, the fluorescence intensity of multiple organs was measured in mice at 4, 24 and 96 h after injection, following which the mice were sacrificed and the organs were harvested.

A drug release study showed a high initial burst (about a third released in the initial 12 h) followed by gradual release (over 84 h). The acidic extracellular matrix in tumor cells promises faster release. NCs were found in all organs that were examined in vivo (Figure 11).

**Figure 12:**
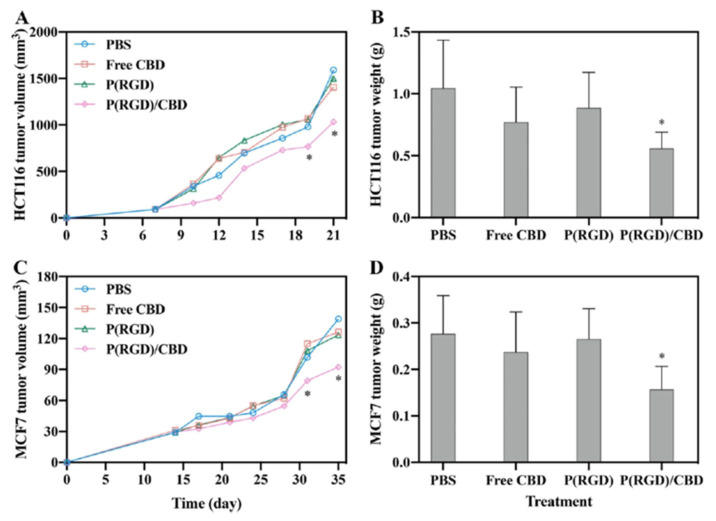
Tumor parameters in nude mouse xenograft model in HCT116 (**A**,**B**) and MCF7 (**C**,**D**) cells after 14 days of bi/triweekly IV injection of PBS, CBD and P(RGD) NCs [9]. * Student’s t-test *p* < 0.05, error bars: standard deviations.

Figure 12 shows targeting, based on the high affinity of RGD to blood vessels and tumor receptors, and treatment of breast cancer and CRC. The targeted delivery significantly increased the efficacy, permitting lower CBD concentrations.

### 5.4. Conjugation of TRAIL

Tumor necrosis factor (TNF)-related apoptosis-inducing ligand (TRAIL) belongs to the TNF cytokine family, which induces apoptosis [56]. Hadad et al. recently conjugated TRAIL to the surface of hollow P(RGD) NCs [11]. Encapsulation of Dox thereby allows targeted dual cancer therapy.

Dox was encapsulated within P(RGD) NCs during the self-assembly process of the proteinoids. The encapsulation and targeted delivery of the drug should reduce its side effects such as cardiac damage and hair loss. TRAIL was protected from rapid enzyme degradation by covalent conjugation [56].

**Figure 13:**
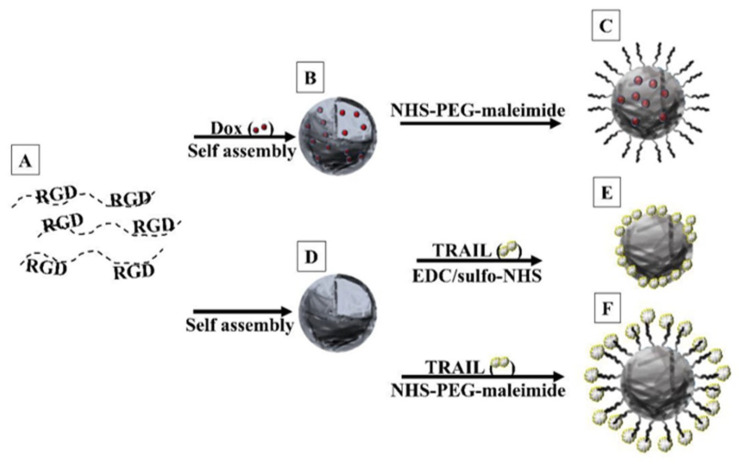
Formation of P(RGD) NCs. (**A**) Polymers are self-assembled (**B**) with Dox and (**C**) PEGylated or (**D**) conjugated to TRAIL (**E**) directly or (**F**) indirectly via PEG (3500 kDa) [12].

Polymers (Figure 13A) were self-assembled to form hollow (Figure 13D) and Dox-loaded (Figure 13B) and PEGylated NCs (Figure 13C). TRAIL was conjugated directly (Figure 13E) and indirectly using a spacer (Figure 13F). The NCs were characterized to determine the optimal method of conjugation. SEM confirmed the preservation of the NCs (shape and dimensions), and ELISA allowed quantitation of TRAIL binding.

PEG was conjugated covalently to the NC surface, and its effect on Dox cytotoxicity and TRAIL release in ovarian cancer (CAOV-3) cells was studied. The hollow NCs were non-toxic, while all encapsulating/conjugated NPs were toxic and showed controlled release. Moreover, in vitro experiments demonstrated that Dox/P(RGD) and TRAIL-P(RGD) NCs were as effective as free Dox/TRAIL (viability of 2 and 9%), while PEGylation considerably reduced the activity (to 20 and 41 %), allowing extended release over several days.

The TRAIL-conjugated drug-loaded NCs are promising for treatment of ovarian cancer. Dox may be applied more safely, and TRAIL stability is increased, while retaining the efficiency of both agents.

### 5.5. Fluorescent NCs Containing Synergistic Drugs

Recently, efficacy/safety of P(RGD) NCs with synergistic drugs targeting tumors was assessed for cancer treatment [18]. Palbociclib (Pal), a CDK4/6 inhibitor, and Alpelisib (Alp), a P13K inhibitor, were co-encapsulated (25 w% of each) with yields of 72% and 95%, respectively. As the drugs have low solubility, different concentrations of Tween 80 were added to the mixed cloudy suspension to obtain a clear solution for drug loading into the NCs. Long-term stability, controlled release and cellular uptake, as well as synergistic cytotoxicity and induced cell death are evident from in vitro experiments.

The hydrodynamic diameter of the hollow NCs, 34 ± 5 nm, decreased following encapsulation of mixed drugs to 22 ± 3 nm. This may be explained by the presence of part of the drugs on the surface of the NCs, preventing water molecules due to their hydrophobic nature from being entrapped and adsorbed. To evaluate this hypothesis, zeta potentials were measured at a pH of 7.5, yielding −6 ± 2 and −0.3 ± 0.8 mV for hollow and drug-loaded NCs, respectively. The negative charge is attributed to aspartic acid on the NC surface. Thus, the increase in the zeta potentials is due to the drug loading and the physical interactions of the drugs with the NC surface, which mask the negative residue.

In vitro cell toxicity was determined by XTT assay on three human cancer cell lines—HCT116 (colon carcinoma), MCF-7 (breast adenocarcinoma) and A549 (lung carcinoma) treated for 24, 48, 72 and 96 h with Cy7-P(RGD) hollow and Pal,Alp-loaded NCs. Cell viability post treatment with the hollow NCs was not significantly different to untreated cells (above 100%), hence the NCs are non-toxic. Treatment with Cy7-P(RGD)/Pal,Alp NCs after 24, 48, 72 and 96 h demonstrated a gradual decrease in cell growth and cell death after 96 h. In contrast, free mixed drugs showed a decrease in cell viability already after 24 h. These results demonstrate the controlled release of the drugs from the NCs compared to the free drugs.

Very recently, NCs were examined in PDX models (colon, breast and gastric cancer) and showed similar results to free drugs with reduced toxicity [57]. One example is the RA-300 PDX model derived from a tumor biopsy of a 50-year-old male patient diagnosed with CRC (well-differentiated adenocarcinoma, stage IV).

**Figure 14:**
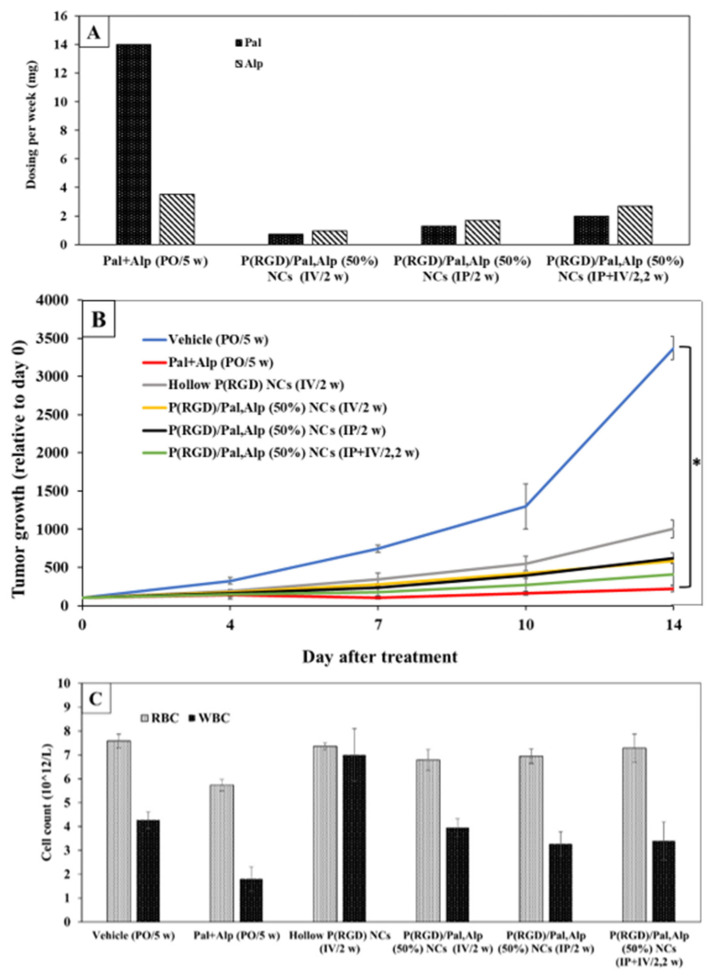
Treatment (14 days) by Pal + Alp and P(RGD) NCs inhibits tumor growth in a PDX colon cancer model (vehicle and drugs administered PO 5 days a week, NCs IP or IV, (**A**)). Tumor growth (**B**) and WBC/RBC at Day 14 (**C**). Data are means with standard errors. * *p* < 0.005 relative to control (5 mice per group) [57].

For the efficacy experiment, the free mixed drugs were administered orally (per os, marked as PO) 5 days a week, whereas the NCs were administered IV and/or intraperitoneally (IP) 2 days per week. As IV injection can lead to damage/blockage of veins, it is advisable to limit the volume and frequency of such injections [58]. Therefore, to achieve the maximum amount of drug, three ways of administering the NCs were tested (IV, IP, or both). IV/IP administration entailed injection twice a week. The vehicle group received no drugs, whereas the free drug group received (PO) a total of 14 mg/week of Pal and 3.5 mg/week of Alp. The three P(RGD)/Pal,Alp (50%) groups included IV (0.72 and 0.95 mg/week of Pal and Alp, respectively), IP (1.3 and 1.7 mg/week, respectively) and IV+IP (2.0 and 2.6 mg/week, respectively), see Figure 14A.

The mice in the vehicle group were sacrificed on Day 14 post treatment due to excessive tumor growth (>1500 mm^3^), while those treated with combined free drugs reached the tumor volume endpoint 2–3 weeks later. Tumor growth (compared to Day 0) is shown in Figure 14B. IV or IP administration led to a similar reduction (2.8-fold) in tumor growth compared to vehicle-treated mice, even though the mice received higher IP doses (2.6-fold). This observation is clinically important, as IP injections are simpler and can be self-administered. Alternating IV and IP administration resulted in a 4-fold reduction in tumor growth at Day 14 relative to the vehicle-treated group, whereas the free drugs demonstrated a 7-fold reduction in tumor growth (Figure 14B). Notably, P(RGD)/Pal,Alp (50%) administered IV + IP had a similar effect as the free drugs, although the doses were lower by 7-fold for Pal and 1.3-fold for Alp.

The high concentrations of free drugs required to achieve a significant inhibitory effect pose a main limitation due to treatment-related toxicity [59]. The most common side effects in patients treated with these drugs include neutropenia, diarrhea, leukopenia and fatigue [60,61]. We therefore evaluated the tolerability of the drug in each treatment group by monitoring the body weight and white/red blood cells (WBC/RBC) (Figure 14C). Treatment with free drugs was associated with a slight decrease in body weight, whereas P(RGD)/Pal,Alp (50%) produced a stable body weight [58]. Reductions in blood count parameters such as WBC and RBC, which are paramount in clinical assessment as they increase the risk of infections and anemia, respectively, were more pronounced with free drugs compared to P(RGD)/Pal,Alp (50%) (Figure 14C). Thus, the results suggest that delivering the combination of drugs by NCs is safer.

There are several studies on the combination of Pal and Alp [59,62], and a clinical study involving this potentially synergistic combination is ongoing [63]. However, to our knowledge, this was the first study investigating this combination using a delivery system that targets the tumor (Pal in combination with other drugs encapsulated in a nanocarrier) [64]. The study provides preclinical in vivo PDX evidence which supports the continued evaluation of P(RGD)Pal,Alp NCs.

## 6. Summary

Thermal step-growth polymerization of suitable amino acids yields a uniform batch of protein-like high molecular weight polymers. Spherical NCs with narrow diameter distribution obtained by self-assembly may encapsulate a variety of molecules including drugs and/or imaging agents.

Proteinoid NCs have great advantages for biomedicine, including non-toxicity, biodegradability, biocompatibility and non-immunogenicity [1,2,3,4,10]. Recent in vitro and in vivo studies that are surveyed in this review report low cost and simple preparation of such NCs from proteinoid polymers (P(EF-PLLA), P(KRHF-PLLA) and P(RGD)) for cancer diagnostics, therapy and theranostics with reduced toxicity.

Amino acids and additives such as PLLA can be tailored for various applications, e.g., diagnostics and therapy. Introducing PLLA into the proteinoid backbone resulted in a proteinoid that was stable and augmented the hydrophobic inner region, leading to the formation of smaller, hollow NCs. Encapsulation of NIR ICG within NCs show promising potential for in vivo diagnosis, due to low background auto-fluorescence and ability to penetrate deep into biomatrices. Another NC used for therapy is P(KRHF-PLLA) NCs which encapsulate Dox and show successful conjugation of PEG to improve their stability and prevent drug leakage. These studies led us to investigate theranostic NCs which co-encapsulate anti-cancer drugs and fluorescence dye as well as specific delivery to tumors by using RGD NCs [12,13].

P(RGD) can serve a dual purpose as a drug carrier through drug encapsulation and a targeting delivery system due to the presence of the RGD sequence on the proteinoid shell. This holds promise for reduced side effects [18]. The delivery system enables targeted transportation to the site of action, reducing the impact on essential tissues and minimizing unwanted side effects [18,57]. In addition, the system shields drugs from rapid degradation or clearance, amplifying their concentration in target tissues, and potentially allowing for lower dose [57].

The terminal amines on the surface of the P(RGD) NCs can be used for conjugation of bioactive compounds. Another potential therapeutic and targeted method was formed by conjugating TRAIL to hollow P(RGD) NCs. The use of Dox/P(RGD) and TRAIL-P(RGD) holds significant potential for targeted cancer therapy, including the possibility of dual therapy that combines the benefits of reduced side effects of Dox and the increased stability of TRAIL. Additionally, RGD proteinoids may have the potential to treat other medical conditions beyond cancer.

The simple way of obtaining NCs which are stable over time enables encapsulating various drugs and/or dyes. In vitro and in vivo experiments indicate that the NCs are capable of penetrating various types of cells, being taken up by different kinds of cells such as breast, colon, glioma and lung cancer cells, and even crossing the blood–brain barrier [9]. Proteinoid-based nanocarriers clearly hold great potential for diagnostic, therapeutic and theranostic applications towards cancer and other indications. Very recent in vivo work in our laboratories with mice illustrated that acidic proteinoid NCs are stable in the stomach, efficiently cross the blood–brain barrier (BBB) and can be administered orally for cancer targeting. Our main future efforts are to illustrate the use of these fluorescent and non-fluorescent NCs for brain cancer theranostics.

## Figures and Tables

**Table 1:** Various proteinoid polymers and their amino acid content ^a^.

Proteinoid Polymer	Amino Acid Content	Main Component of Amino Acids ^a^	Segment
**P(EF-PLLA)**	L-glutamic acid L-phenylalanine	L-glutamic acid	Poly-L-lactic acid (PLLA)
**P(KRHF)**	L-lysine L-arginine L-histidine L-phenylalanine	L-lysine	PLLA
**P(RGD)**	D-arginine L-glycine L-aspartic acid	L-aspartic acid	------

^a^ A tri-functional amino acid—Glu/Asp/Lys—is a main component, providing a solvent by cyclization and serving as an initiator.

## Data Availability

Not applicable.
